# Attenuation of microglial activation in a mouse model of Alzheimer’s disease via NFAT inhibition

**DOI:** 10.1186/s12974-015-0255-2

**Published:** 2015-03-04

**Authors:** Lalida Rojanathammanee, Angela M Floden, Gunjan D Manocha, Colin K Combs

**Affiliations:** Institute of Science, Suranaree University of Technology, 111 University Avenue, Suranaree Subdistric, Nakhon Ratchasima, 30000 Thailand; Department of Basic Sciences, University of North Dakota School of Medicine and Health Sciences, 504 Hamline Street, Neuroscience Building, Grand Forks, ND 58203 USA

**Keywords:** Alzheimer, NFAT, Amyloid, Microglia

## Abstract

**Background:**

Amyloid β (Aβ) peptide is hypothesized to stimulate microglia to acquire their characteristic proinflammatory phenotype in Alzheimer’s disease (AD) brains. The specific mechanisms by which Aβ leads to microglial activation remain an area of interest for identifying attractive molecular targets for intervention. Based upon the fact that microglia express the proinflammatory transcription factor, nuclear factor of activated T cells (NFAT), we hypothesized that NFAT activity is required for the Aβ-stimulated microgliosis that occurs during disease.

**Methods:**

Primary murine microglia cultures were stimulated with Aβ in the absence or presence of NFAT inhibitors, FK506 and tat-VIVIT peptide, to quantify secretion of cytokines, neurotoxins, or Aβ phagocytosis. A transgenic mouse model of AD, APP/PS1, was treated subcutaneously via mini-osmotic pumps with FK506 or tat-VIVIT to quantify effects on cytokines, microgliosis, plaque load, and memory.

**Results:**

Expression of various NFAT isoforms was verified in primary murine microglia through Western blot analysis. Microglial cultures were stimulated with Aβ fibrils in the absence or presence of the NFAT inhibitors, FK506 and tat-VIVIT, to demonstrate that NFAT activity regulated Aβ phagocytosis, neurotoxin secretion, and cytokine secretion. Delivery of FK506 and tat-VIVIT to transgenic APP/PS1 mice attenuated spleen but not brain cytokine levels. However, FK506 and tat-VIVIT significantly attenuated both microgliosis and Aβ plaque load in treated mice compared to controls. Surprisingly, this did not correlate with changes in memory performance via T-maze testing.

**Conclusions:**

Our findings suggest that development of specific NFAT inhibitors may offer promise as an effective strategy for attenuating the microgliosis and Aβ plaque deposition that occur in AD.

**Electronic supplementary material:**

The online version of this article (doi:10.1186/s12974-015-0255-2) contains supplementary material, which is available to authorized users.

## Background

Alzheimer’s disease is characterized by the presence of reactive microglia associated with amyloid β (Aβ) peptide-containing plaques [1-4]. Since Aβ oligomers and fibrils can serve as ligands for microglial activation, they are often hypothesized to contribute to the proinflammatory phenotype of microglia during disease [5-21]. In addition, the Aβ peptides have a characterized ability to stimulate dysregulated intracellular calcium homeostasis, in neurons and glia, which can lead to activation of the calcium-dependent phosphatase, calcineurin [13,22-34]. Calcineurin activity changes have been implicated in Alzheimer’s disease (AD) through a variety of mechanisms including high expression and activity in the hippocampus, calcineurin-mediated synaptic loss, neuronal death, impaired cognition, Aβ production, and astrocyte activation [22,23,35-42].

One of the most abundant and well-studied substrates of calcineurin is the transcription factor, nuclear factor of activated T cells, NFAT. Although its biology has been more classically described in the immune system [43], numerous isoforms of NFAT are expressed in the brain [44-48]. In order for it to become activated, NFAT is dephosphorylated by calcineurin for translocation to the nucleus leading to altered immune cell behavior and cytokine expression working in conjunction with numerous other transcription factors including AP-1 and NFκB [43,49-59]. In AD brains, various isoforms of NFAT demonstrate differential activation profiles with nuclear fractions of NFAT1 (also known as NFATc2) elevated in patients with mild cognitive impairment (MCI) while nuclear fractions of NFAT3 (NFATc4) are increased in AD patient brains [38]. In addition, increased levels of the cytokines, tumor necrosis factor-α (TNFα), interleukin-1 beta (IL-1β), and granulocyte-macrophage colony-stimulating factor (GM-CSF), positively correlate with the nuclear fraction of NFAT1 in both MCI and AD tissue, thus implicating NFAT1 in neuroinflammation during early stages of AD-related cognitive decline [38]. NFAT3 has also been shown to be involved in neurodegenerative processes such as neuronal cell death [46,60]. Collectively, these reports suggest that a sequence of events involving Aβ-stimulated or calcium dysregulation-mediated calcineurin/NFAT activation and subsequent neuronal or glial changes represents an attractive response pathway for therapeutic targeting in AD.

Because extensive work has been done to define astroglial [38,42,47,48,61-66] and neuronal [22,23,41,44-46,67] NFAT functions with regard to regulating proinflammatory, neurodegenerative, apoptotic, sprouting, or survival responses in the brain, we have chosen instead to examine NFAT biology in the brain resident immune cells, microglia. It is clear that microglia express NFAT isoforms [65,68-72] with particularly high levels of NFATc2 [70,71]. In addition, microglial stimulation increases NFAT activity and proinflammatory changes which can be attenuated using NFAT inhibitors such as VIVIT peptide [38,42,48,63,70,73-77]. Based upon our prior work demonstrating a role for NFAT in regulating microglial response to Aβ stimulation [71], we continue, in this study, to define a contribution of microglial NFAT to inflammatory changes in AD using both *in vitro* microglial cultures as well as the APP/PS1 transgenic mouse model of disease. We used two different NFAT activation inhibitors, the clinically available small molecule FK506 [78,79] and the cell penetrant tat-VIVIT peptide [70], to understand the effect of NFAT inhibition on microglial phenotype.

## Methods

### Antibodies and reagents

Anti-NFATc1 (NFAT2), anti-NFATc4 (NFAT3), anti-NFATc3 (NFAT4), anti-phosphoNFATc2 (NFAT1), anti-ERK2, and α-tubulin antibodies were purchased from Santa Cruz Biotechnology (Santa Cruz, CA). The anti-MAP2 antibody was from Cell Signaling Technology Inc (Danvers, MA, USA). HRP-conjugated secondary antibodies were also obtained from Santa Cruz Biotechnology (Dallas, TX, USA). The anti-NFATc2 (NFAT1) antibody and FK506 were obtained from Abcam (Cambridge, MA, USA). Anti-CD68 antibody was from AbD Serotec (Raleigh, NC, USA). Anti-APP antibody was from Zymed Laboratories (San Francisco, CA, USA). Elite Vectastain ABC Avidin and Biotin, Vector VIP, biotinylated anti-rat and anti-mouse antibodies were obtained from Vector Laboratories Inc (Burlingame, CA, USA). Synaptophysin antibody was purchased from Chemicon International, Inc (Temecula, CA, USA). Phospho-NFκB (p65) antibody, NFκB (p65) antibody, PSD95 antibody, and beta site APP cleaving enzyme (BACE) antibody were from Cell Signaling Technology Inc (Danvers, MA, USA). Anti-Aβ (4G8) antibody was from Covance (Emeryville, CA, USA). The c-Fos antibody was purchased from Novus Biologicals, LLC (Littleton, CO, USA). Lipopolysaccharide (LPS) and MTT were purchased from Sigma-Aldrich (St. Louis, MO, USA). Human Aβ1-42 was purchased from Bachem (Torrance, CA, USA) and fibrillized before use according to our prior protocol [80]. A VIVIT peptide sequence, MAGPHPVIVITGPHEE, that interacts with NFAT at its calcineurin binding site and inhibits its activation was used along with its negative control, VEET, peptide [73]. A cell-permeable variation of VIVIT peptide, tat-VIVIT, fused with an HIV-1 TAT protein cationic transduction domain [76,77], was generated to examine the potential of this novel peptide construct for NFAT inhibition as in our prior work [70]. The inhibitory peptide tat-VIVIT (H-YGRKKRRQRRR-AA-MAGPHPVIVITGPHEE-NH_2_) and negative control peptide, tat-VEET (H-YGRKKRRQRRR-AA-MAGPPHIVEETGPHVI-NH_2_), were synthesized by Dr. Satya Yadav at the Molecular Biotechnology Core Laboratory at the Cleveland Clinic Foundation (Cleveland, OH). Cytokine enzyme-linked immunosorbent assay (ELISA) kits for TNFα, interleukin-6 (IL-6), IL-1β were purchased from R&D Systems (Minneapolis, MN, USA). Fluorescein isothiocyanate (FITC) bioparticles were purchased from Invitrogen (Carlsbad, CA, USA).

### Animals

All animal use was approved by the University of North Dakota Institutional Animal Care and Use Committee (UND IACUC). The Jackson Laboratory (Bar Harbor, ME) transgenic mouse line, strain 005864 B6.Cg-Tg (APPswe,PSEN1dE9)85Dbo/J, and their wild-type littermate controls were used for this study. Mice were provided food and water *ad libitum* and housed in a 12-h light:dark cycle. The investigation conforms to the National Research Council of the National Academics Guide for the Care and Use of Laboratory Animals (eighth edition). These APP/PS1 transgenic mice express the human *APP*, Aβ precursor protein (A4) with the Swedish mutations K595N/M596L, and the human *PSEN1*, presenilin 1 with the DeltaE9 mutation under control of the mouse prion promoter.

### Cell culture

Primary microglia were derived, as described previously [8], from the brains of postnatal day 1 to 3 wild-type C57BL/6 wild type mice. Briefly, meninges-free cortices were removed, trypsinized, and triturated in microglia media (DMEM/F12 media containing L-glutamine (Invitrogen, Carlsbad, CA, USA) and 20% heat-inactivated FBS) and placed in T-75 flasks. Media in the flasks was replaced completely after 24 h and partially after 7 days with fresh media. Cells were harvested, counted, and used at day 14 *in vitro*.

Neurons were cultured from cortices of wild-type embryonic day 16 (E16) mice (C57BL/6). Meninges-free cortices were isolated, trypsinized, and plated onto poly-L-lysine-coated tissue culture wells containing Neurobasal media with L-glutamine and B27 supplements (Invitrogen, Rockville, MD, USA). Neurons were allowed to grow in the media for 7 days *in vitro* in order to provide >95% purity.

Jurkat cells were obtained from the American Type Culture Collection (ATCC, Manassas, VA, USA) and maintained in RPMI-1640 medium (Gibco RBL, Rockville, MD, USA) containing 10% heat-inactivated fetal bovine serum (US Biotechnologies Inc., Parkerford, PA, USA), 5 mM HEPES, and 1.5 μg/mL antibiotics (penicillin/streptomycin/neomycin).

### Cell stimulations

To determine the levels of proinflammatory cytokines, cells were pretreated with FK506 or tat-VIVIT for 30 min followed by 10 μM Aβ 1-42 stimulation for 24 h. Media was collected for ELISA.

In order to understand the effect of NFAT inhibition on neuronal cell viability, 7-day *in vitro* primary neurons were grown in microglial conditioned media for 72 h as previously described [25]. Briefly, 96-well cell culture plates were coated with Aβ 1-42 fibrils (48 pmole/mm^2^) in the presence or absence of 0.1 μM FK506 and 1 μM FK506 in serum-free Neurobasal media with B27 supplements. Microglia cells were plated on the surface-bound Aβ fibrils for 48 h. Following the stimulation, media from wells with only Aβ (negative control), wells with only microglia, and conditioned media from wells with microglia stimulated with Aβ were transferred to neurons for 72 h. Neurons were also left untreated or treated with 0.1 μM FK506 or 1 μM FK506 added to the neurons along with the conditioned media. Following all the treatments, neurons were fixed with 4% paraformaldehyde and immunostained with anti-MAP2 antibody. As in our prior work [25], surviving cells immunostained positive for MAP2 were counted, averaged, and plotted (±SD). Stimulations were performed in duplicate and repeated three times. A counting grid was placed over the wells to count the number of neurons from eight identical fields for each condition.

### ELISA

The levels of proinflammatory cytokines TNFα, IL-6, and IL-1β were measured using ELISA. Media from cell stimulation experiments was transferred onto ELISA plates and levels measured using the manufacturer’s protocol. For brain and spleen ELISA, flash-frozen hippocampi or spleen tissue was lysed in radioimmunoprecipitation assay (RIPA) buffer and lysates were centrifuged at 14,000 rpm, 4°C, for 10 min to remove any insoluble material. Supernatants were then transferred onto the ELISA plates and cytokine levels were measured and averaged (±SD) as per the manufacturer protocol.

### MTT reduction assay

Cell viability was measured using the MTT reduction assay. After 24-h stimulation with Aβ in the presence or absence of FK506 or tat-VIVIT, cells were treated with 0.1 mg/mL MTT (3-(4,5-dimethylthiazol-2-yl)-2,5-diphenyltetrazolium bromide) for 4 h at 37°C. The media was aspirated and precipitated formazan dissolved with isopropanol. Optical density was measured at 560 nm, averaged (±SD), and plotted.

### Phagocytosis assay

In order to determine the phagocytic ability of Aβ-stimulated microglia in the presence or absence of NFAT inhibitors, microglia were plated onto 96-well culture plates and were either untreated or treated with 1 μM FK506 for 24 h. Cells were then treated with either 500 nM FITC-conjugated Aβ1-42 fibrils or 0.25 mg/mL FITC-conjugated bioparticles for an additional 6 h. After incubation, the media was removed and fluorescence from extracellular peptide or membrane-bound bioparticles was quenched via a rinse with 0.25% trypan blue. Fluorescence intensity from phagocytosed peptide and bioparticles was measured using a fluorescent plate reader (480-nm excitation and 520-nm emission) and averaged (±SD).

### Animal treatments

NFAT inhibitors FK506, tat-VIVIT, and tat-VEET were infused subcutaneously into male APP/PS1 mice at 12 months of age. The compounds were delivered via mini-osmotic pumps (model 1004, 0.11 μL/h delivery rate for 28 days, Alzet, Cupertino, CA, USA). Pumps delivered either vehicle (DMSO/HEPES) (*n* = 8), FK506 (1 mg/kg/day) (*n* = 7), VIVIT (0.5 mg/kg/day) (*n* = 8), or negative control scrambled peptide, VEET (0.5 mg/kg/day) (*n* = 8), for 28 days. At the end of the infusion period, mice were behaviorally tested (day 28 of 28-day infusion) then euthanized and perfused with PBS-CaCl_2_, and brains and spleens were rapidly collected. Control untreated APP/PS1 mice were collected at a comparable age of completion, 13 months (*n* = 6). The right hemispheres of the brains were fixed in 4% paraformaldehyde, and the left hemispheres were flash frozen in liquid nitrogen for biochemical analysis.

### Western blots

Temporal cortices were dissected out of the left brain hemispheres from the control, the vehicle-treated, or the inhibitor-infused mice and lysed, sonicated in RIPA buffer, and quantitated using the Bradford method [81]. The lysates were resolved on 10% SDS-PAGE and transferred to polyvinylidene difluoride membranes for Western blotting using anti-APP, anti-BACE, anti-synaptophysin, anti-PSD95, anti-pNFκB (p65), and anti-pNFATc2 antibodies with anti-α-tubulin, NFκB (p65) (for pNFκB), and NFATc2 (for pNFATc2) antibodies used for loading controls. Western blots were quantified using Adobe Photoshop software. Optical densities (O.D.) of bands were normalized against their respective loading controls and averaged (±SD).

### Immunohistochemistry

Right hemispheres from brains collected from untreated or NFAT inhibitor-infused mice were fixed in 4% paraformaldehyde and cryoprotected in 30% sucrose (in 0.1 M phosphate buffer) by replacing the sucrose solution every 3 to 4 days at least three times. The fixed tissue was then embedded in a 15% gelatin matrix, and the gelatin block again fixed in 4% paraformaldehyde for 3 to 4 days followed by cryoprotection in 30% sucrose solution. The block was then flash frozen using dry ice/isopentane and serially cut (40 μm) on a freezing microtome. Serial sections were used for immunostaining using anti-Aβ (4G8) and anti-CD68 (macrophage/microglia marker) antibodies using Vector VIP as the chromogen. In order to quantitate immunostaining, ×1.25 pictures of three consecutive sections (960 μm apart) throughout the temporal cortex region were taken. Optical densities from hippocampi from each brain section were measured using Adobe Photoshop software. The optical density values per condition (five conditions), per brain (six to eight brains per condition) and per section (three sections per brain) were obtained, averaged (±SD), and plotted.

### T-maze

T-maze analysis was performed as described by Wenk [82]. Briefly, on the 28th day after initiation of infusion, the mice were placed into the starting arm of the T-maze and the door was raised to allow the mice to walk down the stem and enter an arm. Once a mouse entered an arm with all four paws, it was returned to the starting arm. The door was closed for 30 s, then raised, and the mouse was allowed to move freely until it entered an arm again. The process was repeated for nine trials and the choice of arms that mice entered was noted. The number of alternations per mouse in each condition was averaged (±SD) and plotted.

### Statistical analyses

Data are presented as mean values ± SD. Statistical significances were calculated using one-way ANOVA with Tukey-Kramer’s *post hoc* comparison and considered significant when *p* values were <0.05.

## Results

### Microglia expressed multiple NFAT isoforms

**Figure 1:**
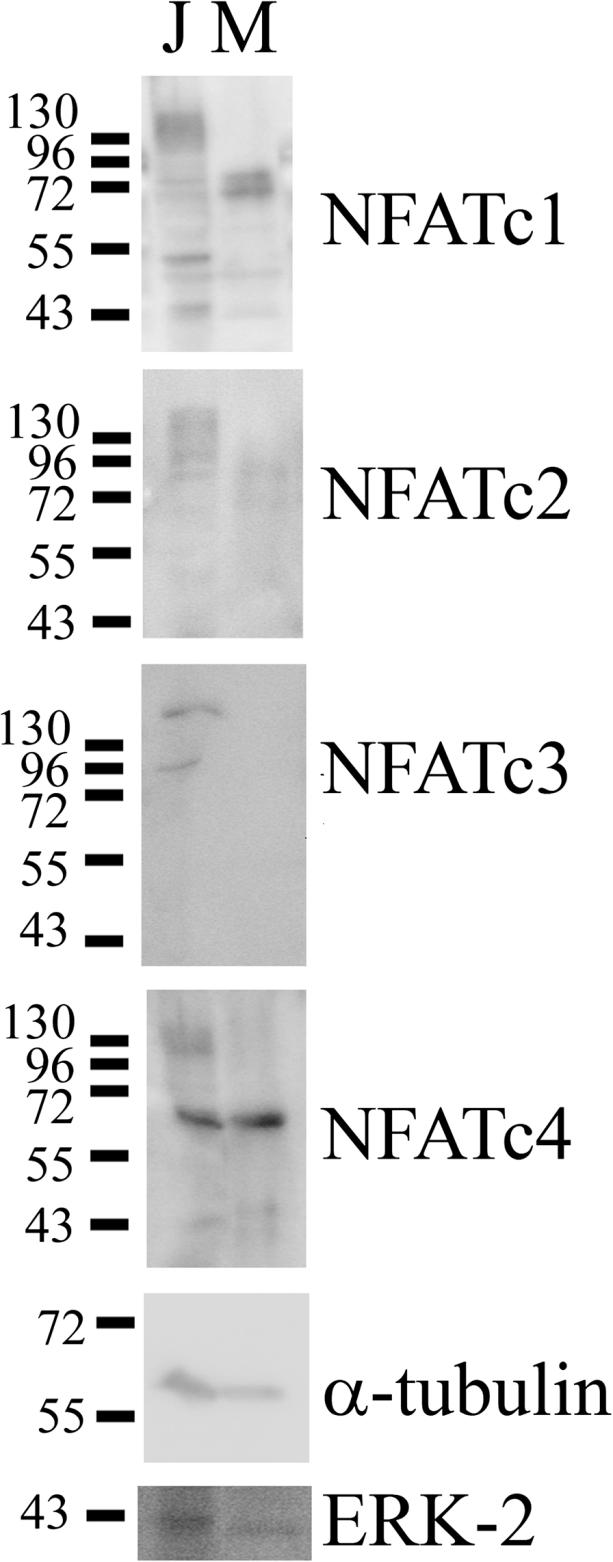
**Microglia expressed multiple NFAT isoforms.** Microglia were grown as mixed glial cultures from postnatal day 0 C57BL6/J mice and purified at day 14 *in vitro*. Lysates of microglia (M) were compared to lysates from the Jurkat human T cell line (J) via Western blot using anti-NFATc1, c2, c3, c4, α-tubulin, and ERK-2 (loading control) antibodies. Multiple bands per NFAT isoform likely reflect the multiple alternative transcripts reported for each isoform. Blots are representative of three independent experiments.

In order to compare microglia expression of NFAT to peripheral immune cells, we compared primary murine microglia to the human T cell line, Jurkat. Based upon the fact that NFAT isoforms in both humans and mice each demonstrate a multitude of alternative splice patterns, we expected a diverse range of protein expression patterns per isoform [83]. As predicted, Western blot analysis demonstrated that Jurkat cells expressed multiple molecular weight NFAT isoforms (Figure 1). Although primary murine microglia also displayed detectable amounts of numerous molecular weight species, only NFATc1, NFATc2, and NFATc4, and not NFATc3, were detectable (Figure 1).

### Microglial NFAT activity was required for increased cytokine secretion following stimulation

**Figure 2:**
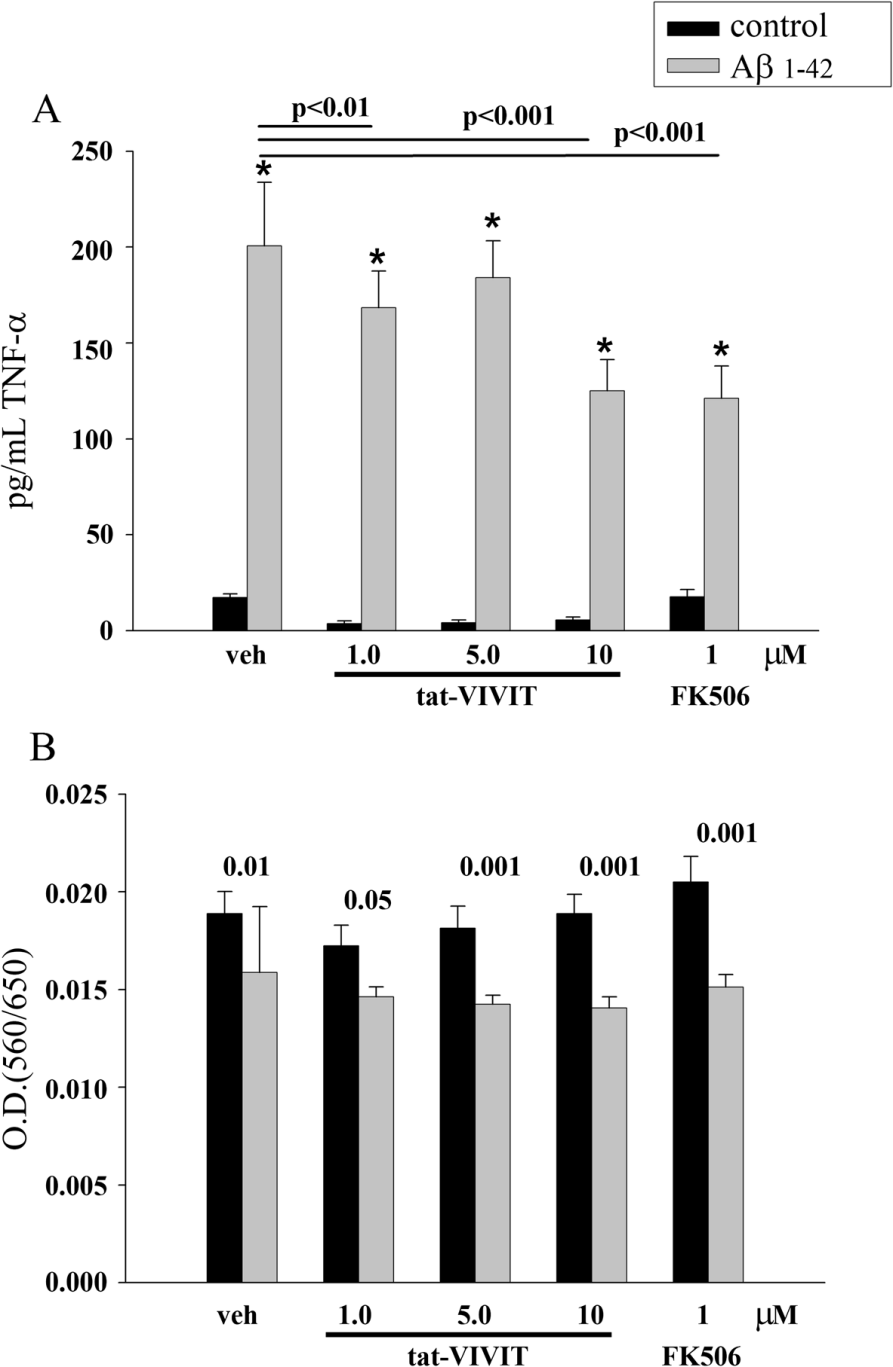
**FK506 and tat-VIVIT attenuated Aβ-stimulated TNFα secretion by microglia.** Microglia were stimulated with 10 μM Aβ1-42 fibrils for 24 h in the absence or presence of 1 μM FK506 or 10 μM tat-VIVIT. **(A)** Media aliquots were taken and ELISAs were performed to quantitate levels of secreted TNFα. **(B)** Media was also taken to perform LDH release assays to quantitate cell death. Conditions were performed with eight repeats and averaged (±SD). Graphs are representative of three independent experiments (*p < 0.001 from control).

In order to determine whether increased NFAT activity was required for altered protein expression in microglia, we next collected media from microglia stimulated 24 h with Aβ or LPS in the absence or presence of tat-VIVIT or FK506 treatments to inhibit NFAT [70,73]. ELISAs for TNFα and interleukin-6 (IL-6) were performed from the media to quantitate any changes in cytokine secretion. Although Aβ treatment alone had an expected slight decrease in cell viability, neither tat-VIVIT nor FK506 further decreased survival (Figure 2). On the other hand, both tat-VIVIT and FK506 significantly attenuated both Aβ- and LPS-stimulated secretion of either cytokine (Figure 2).

### NFAT activation increased microglial uptake of fibrillar Aβ

**Figure 3:**
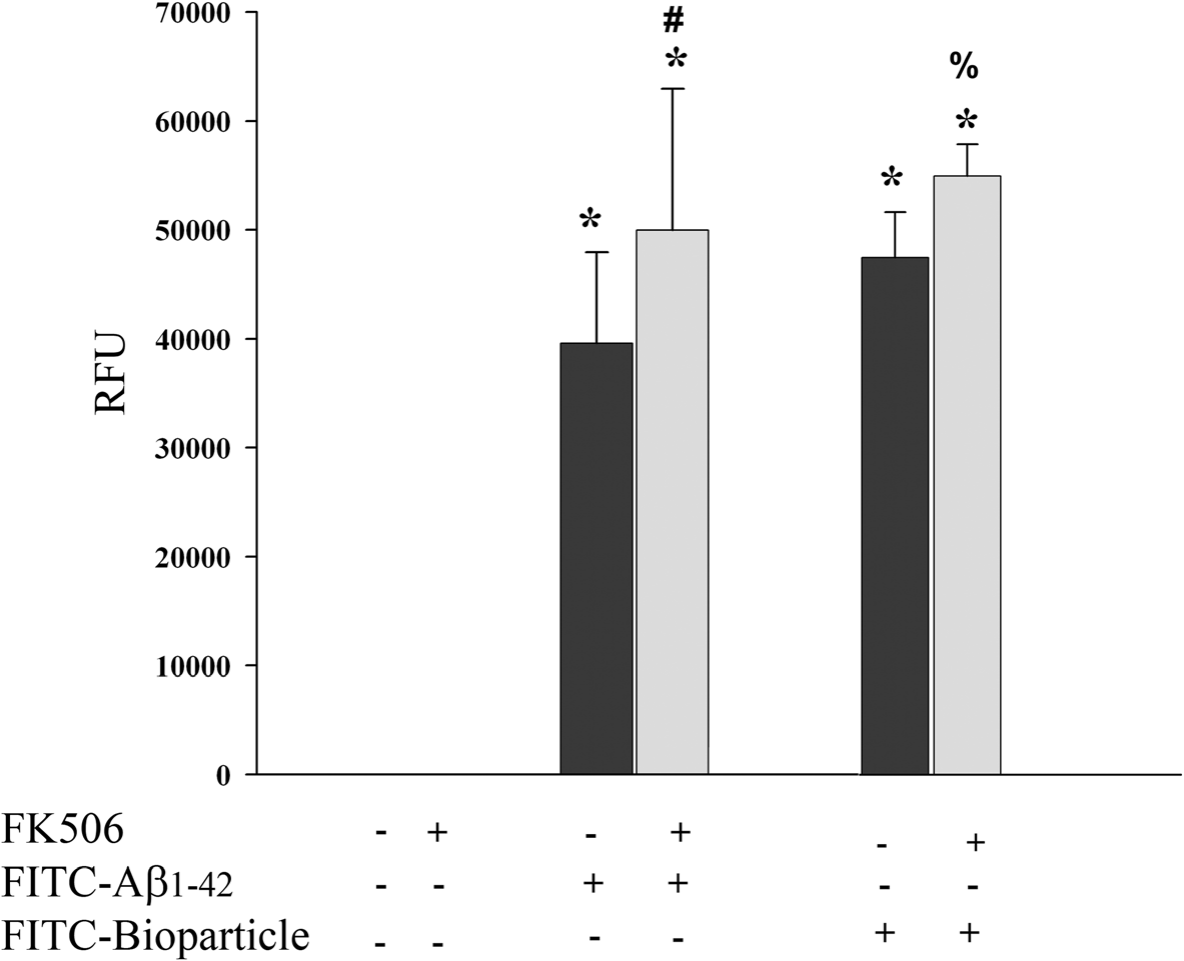
**FK506 stimulated increased microglial uptake of Aβ fibrils.** Microglia were plated in the absence or presence of 1 μM FK506 for 24 h. Either 500 nM FITC-conjugated Aβ1-42 fibrils or 0.25 mg/mL FITC-conjugated bioparticles were added to the cells for an additional 6 h. After incubation, the media was removed and the signal from unphagocytosed peptide was quenched with trypan blue. Fluorescence intensity from phagocytosed peptide and bioparticle was quantitated using a fluorescent plate reader (480-nm excitation and 520-nm emission) and averaged (±SD). Conditions were performed with eight repeats per condition and graphs are representative of three independent experiments (±SD) (*p < 0.001 from control, ^#^ *p* < 0.05 from Aβ, ^%^ *p* < 0.001 from bioparticle).

Decreasing proinflammatory secretion from microglia is arguably an advantageous condition in the AD brain, but decreasing phagocytic ability in parallel may lead to an undesirable increase in fibrillar Aβ plaque deposition. To determine whether NFAT inhibition attenuated microglial ability to clear fibrillar Aβ via phagocytic uptake, microglia were stimulated with FK506 and uptake of FITC-conjugated Aβ1-42 fibrils was quantified. In contrast to the inhibitory consequence of FK506 treatment with regard to cytokine secretion, drug treatment actually increased phagocytic uptake of Aβ fibrils (Figure 3). Moreover, this was not limited to Aβ. Phagocytosis of FITC-labeled bioparticles was also increased following FK506 treatment suggesting that the benefits were not specific to a particular particle type (Figure 3).

### NFAT inhibition attenuated Aβ fibril-stimulated microglial-mediated neuron death *in vitro*

**Figure 4:**
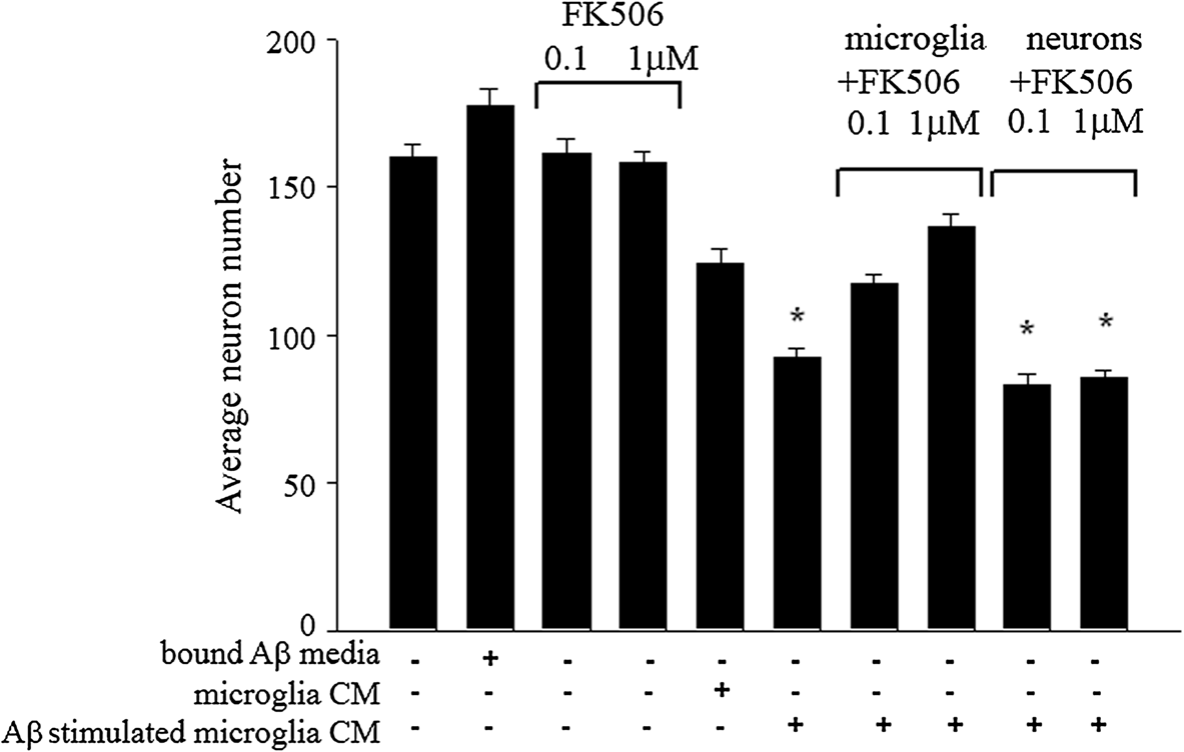
**FK506 prevented microglial generation of neurotoxic conditioned media following Aβ stimulation.** Microglia were stimulated 48 h in the absence or presence of surface-bound Aβ1-42 fibrils (48 pmole/mm^2^) and 0.1 and 1 μM FK506. Conditioned media from bound Aβ wells alone (negative control), microglia only wells (microglia CM), and Aβ-stimulated microglia wells (Aβ-stimulated microglia CM) +/− FK506 were transferred to 7 day *in vitro* primary mouse cortical neuron cultures for 72 h. In addition, FK506 (0.1 and 1 μM) was added directly to neurons (negative control) and to neurons along with Aβ-stimulated microglia. Neurons were fixed and stained with an anti-MAP2 antibody and surviving cells counted and averaged (±SD). Stimulations were performed in duplicate and repeated three times. Results are the average of three independent experiments (*p < 0.001 from control).

Another demonstrative therapeutic advantage of NFAT inhibition in AD would be evidence of neuroprotection. To generate evidence of whether or not microglial NFAT inhibition could improve neuron survival in AD, we next stimulated microglia with and without Aβ fibrils in the absence or presence of FK506 and transferred this conditioned media to primary cortical neuron cultures. Our prior work has demonstrated that this Aβ-stimulated conditioned media is potently toxic to neuron cultures compared to media from unstimulated microglia [84]. Treatment of the microglia with FK506 attenuated the toxicity of Aβ-stimulated conditioned media (Figure 4). Importantly, this neuroprotection was via microglial inhibition since adding FK506 directly to neurons in the presence of the Aβ-stimulated conditioned media had no ability to offer protection (Figure 4).

### Tat-VIVIT and FK506 attenuated microgliosis in a transgenic mouse model of Alzheimer’s disease

**Figure 5:**
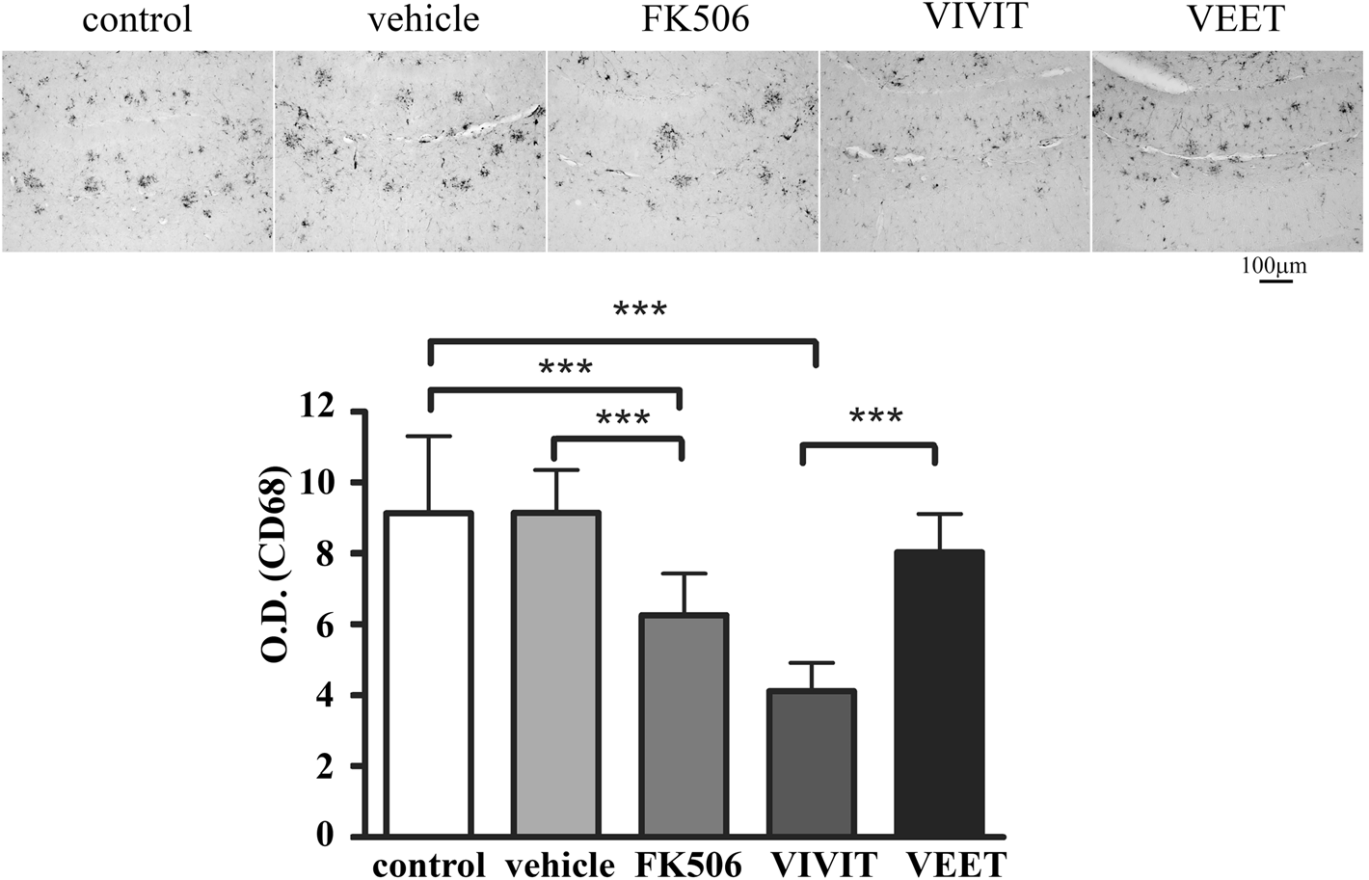
**FK506 and tat-VIVIT decreased CD68 immunoreactivity in APP/PS1 mice.** Twelve-month-old male APP/PS1 mice were treated via subcutaneous delivery for 28 days with no treatment (control), vehicle DMSO, 1 mg/kg/day FK506, 0.5 mg/kg/day VIVIT, or 0.5 mg/kg/day negative control scrambled peptide, VEET (*n* = 6 to 8/condition). Brains were collected, sectioned, and stained using anti-CD68 antibody and staining intensities were quantified from serial sections from the hippocampus. The optical density values per condition (five conditions), per brain (six to eight brains per condition), and per section (three sections per brain) were obtained, averaged (±SD), and plotted (***p < 0.001).

In order to test the efficacy of NFAT inhibition for particularly microglial activation *in vivo*, we next relied on a transgenic mouse model of AD with robust microgliosis [80]. Using an APP/PS1 transgenic mouse model of AD, we tested the efficacy of NFAT inhibition as a therapeutic for AD. APP/PS1 mice were treated, for 28 days via mini-osmotic pumps, with either FK506 or the brain-permeable NFAT inhibitory peptide, tat-VIVIT. Immunostaining for CD68, the microglial marker, demonstrated that both FK506 and tat-VIVIT but not the negative control-scrambled peptide, tat-VEET [74], significantly attenuated CD68 immunoreactivity in the hippocampus of the mice as predicted (Figure 5).

### Tat-VIVIT and FK506 reduced Aβ plaque load in a transgenic mouse model of Alzheimer’s disease

**Figure 6:**
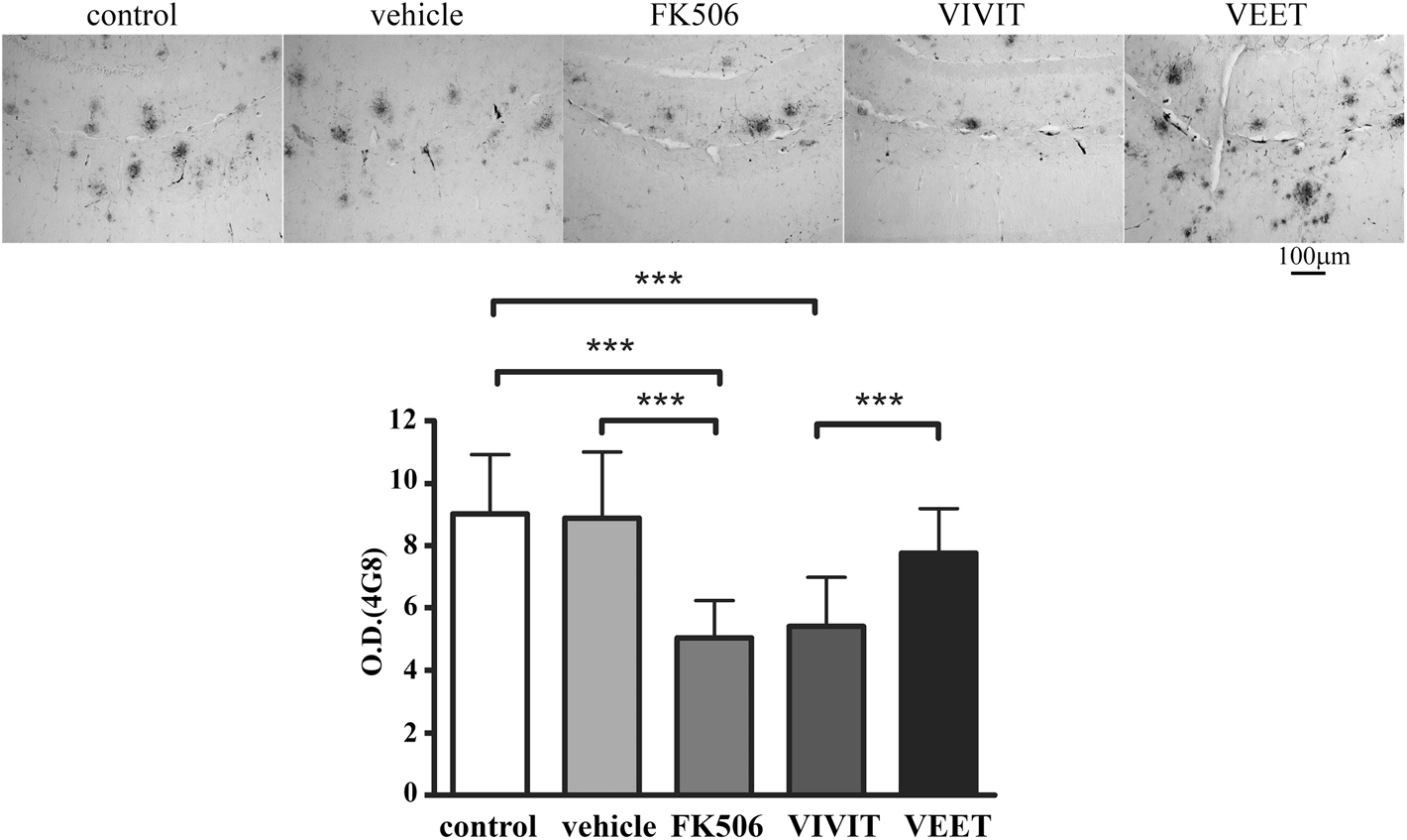
**FK506 and VIVIT decreased Aβ plaque load in APP/PS1 mice.** Twelve-month-old male APP/PS1 mice were treated via subcutaneously delivery for 28 days with no treatment (control), vehicle DMSO, 1 mg/kg/day FK506, 0.5 mg/kg/day VIVIT, or 0.5 mg/kg/day negative control scrambled peptide, VEET (*n* = 6 to 8/condition). Brains were collected, sectioned, and stained using anti-Aβ (4G8) antibody and staining intensities were quantified from serial sections from the hippocampus. The optical density values per condition (five conditions), per brain (six to eight brains per condition), and per section (three sections per brain) were obtained, averaged (±SD), and plotted (***p < 0.001).

In order to determine whether microglial inhibition resulted in any change in plaque load in these mice, Aβ immunoreactive plaques were stained from serial hippocampal sections from the mice using the anti-Aβ antibody, 4G8. Plaque density was quantified to reveal that both FK506 and tat-VIVIT attenuated Aβ peptide-containing plaque load (Figure 6). This suggested that decreasing reactive microglial phenotype via NFAT inhibition had an additional beneficial effect of also decreasing plaque load.

### Tat-VIVIT and FK506 reduced cytokine secretion peripherally in a transgenic mouse model of Alzheimer’s disease

**Figure 7:**
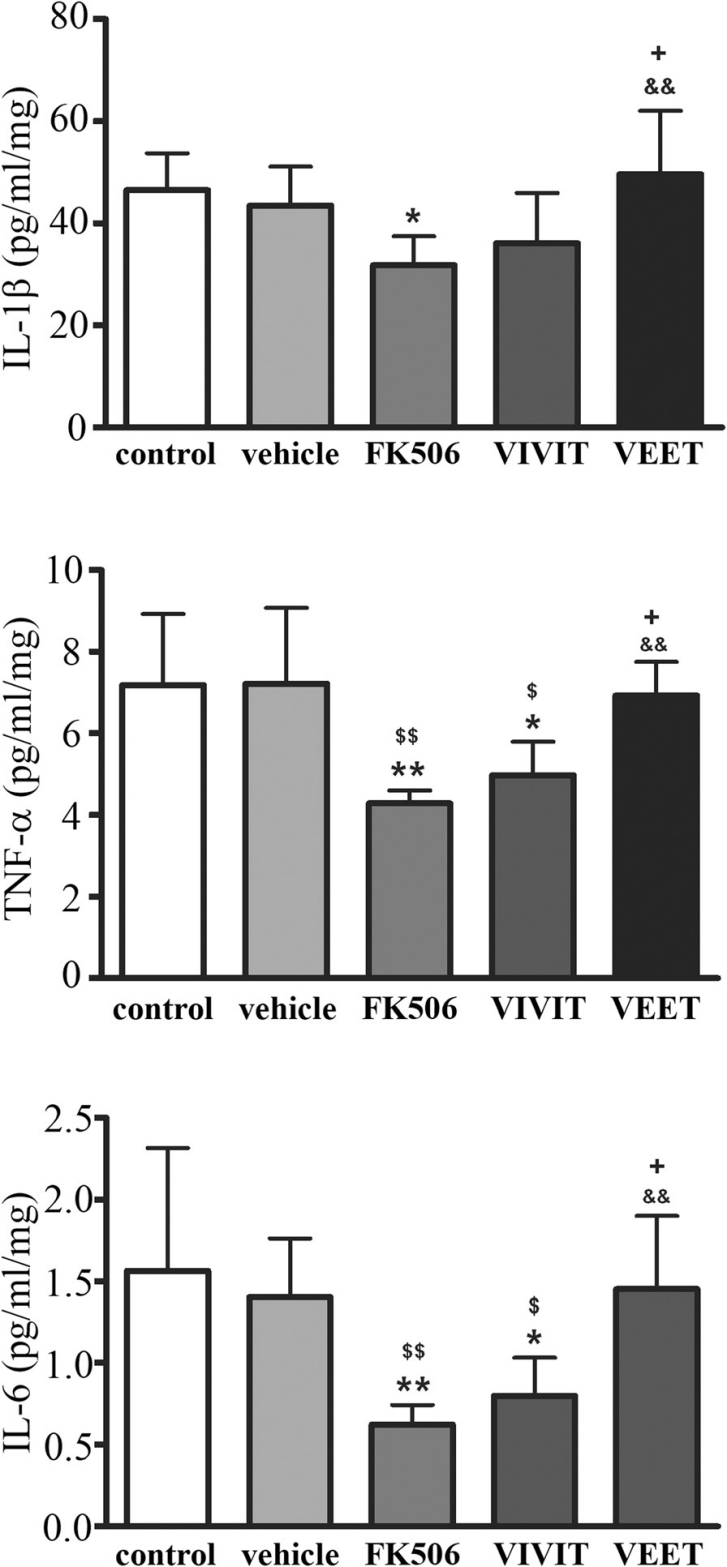
**FK506 and tat-VIVIT decreased spleen cytokine levels in APP/PS1 mice.** Twelve-month-old male APP/PS1 mice were treated via subcutaneous delivery for 28 days with no treatment (control), vehicle DMSO, 1 mg/kg/day FK506, 0.5 mg/kg/day VIVIT, or 0.5 mg/kg/day negative control scrambled peptide, VEET (*n* = 6 to 8/condition). Spleens were collected, lysed, and used for TNFα, IL-1β, and IL-6 ELISAs. (*p < 0.05, **p < 0.01 from control; ^$^ *p* < 0.01, ^$$^ *p* < 0.01 from vehicle; +*p* < 0.05 from VIVIT; &&*p* < 0.01 from FK506)

**Figure 8:**
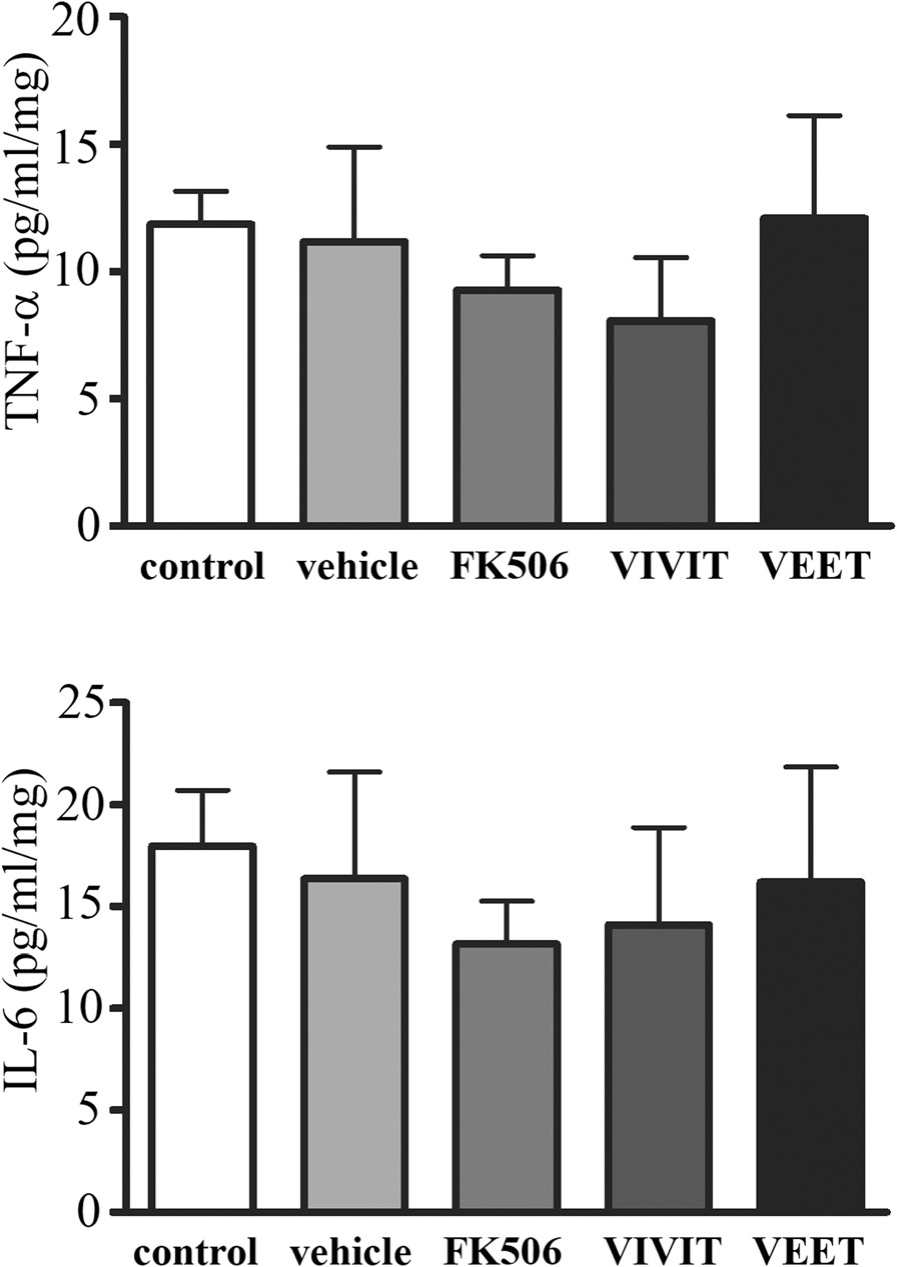
**FK506 and tat-VIVIT did not affect brain cytokine levels in APP/PS1 mice.** Twelve-month-old male APP/PS1 mice were treated via subcutaneous delivery for 28 days with no treatment (control), vehicle DMSO, 1 mg/kg/day FK506, 0.5 mg/kg/day VIVIT, or 0.5 mg/kg/day negative control scrambled peptide, VEET (*n* = 6 to 8/condition). Hippocampi were collected, lysed, and used for TNFα and IL-6 ELISAs.

The brains and spleens of these mice were next used to measure changes in NFAT activity and cytokine levels to determine whether the decreases in microglial and Aβ plaque immunoreactivity correlated with an actual decrease in NFAT activity and anti-inflammatory effects after FK506 or tat-VIVIT treatment. Although FK506 significantly decreased IL-6, TNFα, and IL-1β levels in the spleens of mice, tat-VIVIT treatment significantly attenuated only IL-6 and TNFα levels (Figure 7). Unexpectedly, tat-VIVIT or FK506 treatment had no effect on levels of brain cytokines (Figure 8) in spite of the changes observed in the spleen (Figure 7) and the significant decrease in microglial immunoreactivity observed (Figure 5). This suggests that a higher concentration of either drug may be required for quantifiable brain anti-inflammatory, NFAT inhibitory effects.

In order to continue characterizing the effects of tat-VIVIT or FK506 treatment on the brain, we performed Western blot analyses to quantify any additional differences due to NFAT inhibitor treatment. Although NFATc1 activity was not different between treatment groups, it was possible that other NFAT isoforms were affected by treatments. Active levels of NFATc2 were quantified by measuring any change in phosphorylation state. However, similar to NFATc1 no increase in active, non-phosphorylated NFATc2 resulted from treatments (Additional file 1: Figure S1). To determine whether the decrease in Aβ plaque levels could be due to altered levels of APP or its processing, APP and BACE were quantified. Again, no differences were observed in any treatment (Additional file 1: Figure S1). To examine whether changes in other transcription factors might correlate with the effects we observed, protein levels of c-Fos and phosphorylated, active p65 NFκB were also examined. However, similar to NFATc2, no differences were observed between groups (Additional file 1: Figure S1). Finally, synaptic changes were assessed by quantifying protein levels of the presynaptic marker, synaptophysin, and the postsynaptic marker, PSD95. No differences were observed between groups (Additional file 1: Figure S1).

### FK506 and tat-VIVIT did not improve spatial memory in APP/PS1 mice

**Figure 9:**
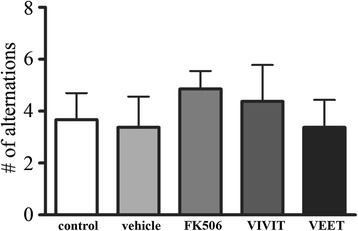
**FK506 and tat-VIVIT did not improve spatial memory in APP/PS1 mice.** Twelve-month-old male APP/PS1 mice were treated via subcutaneous delivery for 28 days with no treatment (control), vehicle DMSO, 1 mg/kg/day FK506, 0.5 mg/kg/day VIVIT, or 0.5 mg/kg/day negative control scrambled peptide, VEET (*n* = 6 to 8/condition). Mice were used for T-maze testing on day 28. Numbers of spontaneous alternations were averaged and graphed (±SD).

In order to determine whether an NFAT inhibition-mediated decrease in microgliosis had an effect on behavior of APP/PS1 mice, we performed T-maze analyses to quantify working memory. Mice infused with tat-VIVIT or FK506 did not show any changes in the performance on T-maze as measured by the number of alternations between the T-maze arms (Figure 9).

## Discussion

Collectively, these results demonstrated that microglia express multiple NFAT isoforms which have a role in regulating the Aβ-stimulated increase in cytokine secretion. Importantly, inhibition of NFAT with tat-VIVIT or FK506 was sufficient to attenuate cytokine secretion and neuron death yet increase Aβ phagocytic uptake *in vitro*. Similarly, peripheral delivery of tat-VIVIT or FK506 to APP/PS1 mice decreased levels of reactive microglia and Aβ immunoreactive plaque load in the brain. However, this did not correlate with changes in either brain NFAT activity or cytokine levels. These findings suggest that development of specific NFAT inhibitors, perhaps independent of the ability to traverse the blood brain barrier, may offer promise for attenuating the microgliosis and Aβ deposition that occur in AD.

Our findings are consistent with numerous other reports suggesting that NFAT activity is a reasonable anti-inflammatory target in AD. It has been shown from AD brain hippocampi that levels of several cytokines, including IL-1β, TNFα, and GM-CSF, are elevated in correlation with nuclear accumulation of NFAT1 (NFATc2) [85]. In addition, Aβ-mediated neurotoxicity is attenuated by NFAT inhibition in APP/PS1 following injection of VIVIT into the cortex [41]. We have found that microglial NFAT may also be important in regulating the complex milieu of the inflammatory environment of the diseased brain. Future work may define whether particular isoforms of NFAT in one cell type versus another are more effective at preventing inflammatory changes.

Although we have shown that FK506 and tat-VIVIT were effective in counteracting Aβ-mediated effects on microglial *in vitro* and attenuating microgliosis in the APP/PS1 line, we appreciate that neither of these agents is NFAT isoform selective. Indeed, FK506 has the added confound of inhibiting calcineurin activity [86]. Therefore, we cannot exclude the possibility that some of the changes we observed from FK506 treatment were due to calcineurin inhibition. Indeed, several others have reported the preclinical benefits of calcineurin inhibition in mouse models of AD [22,42,87-91]. Our intent was to contrast the effects of the tat-VIVIT treatment with those of FK506 due to the fact that the VIVIT peptide has no reported ability to inhibit calcineurin [73,92,93]. In this way, we predicted that it might be possible to determine, to some extent, how much of the anti-inflammatory effects were due to calcineurin versus NFAT inhibition. In addition, it is important to point out that some of the decrease in cytokine secretion we observed in our *in vitro* experiments could be due to a loss of viability from the Aβ treatment itself. However, the dose-dependent inhibitory effect of the VIVIT peptide treatment on TNFα secretion still exists in spite of a similar reduction in Aβ treatment across all conditions. Nevertheless, future work will require a lower, nontoxic concentration of peptide below 10 μM to prevent the confound of toxicity we observed.

It is likely that the concentrations or delivery method of our FK506 and tat-VIVIT for the *in vivo* experiments was not optimum. For example, despite the fact that both FK506 and tat-VIVIT peptide attenuated spleen peripheral cytokine levels, we did not observe the same changes in the brain. Indeed, much higher concentrations have been reported for both FK506 [88,90] and VIVIT peptide [41,63,75] to achieve brain effects. Although FK506 has been used at a dosage of 1 mg/kg/day, similar to our own, to attenuate hippocampal atrophy and microgliosis in a tauopathy mouse model [94], others have reported improved cognitive function and spine density restoration using much higher concentrations of the drug ranging from 10 to 100 mg/kg/day of FK506 [88,90]. Similarly, studies showing effects of VIVIT in inhibiting Aβ-mediated neurodegeneration involve viral vector overexpression of VIVIT in the cortex or hippocampus likely reaching much higher local concentrations than our paradigm [41,63]. A study similar to our own using peptide rather than an expression system for VIVIT demonstrated a role for NFAT in cardiac hypertrophy and involved injecting VIVIT at a dose of 10 mg/kg subcutaneously [75]. Therefore, it is quite possible that the brain concentrations of drug and peptide we achieved were simply not high enough to inhibit NFAT activity during our delivery paradigm. This possibility is consistent with the fact that we were not able to quantify any decrease in active NFATc2 levels in the FK506- and tat-VIVIT-infused mouse brains. In this case, higher drug or peptide concentrations or direct brain delivery might achieve more robust anti-gliotic and anti-inflammatory effects in future work. Although it is not clear what adverse effects would result from higher concentration or more prolonged delivery of the VIVIT peptide, it is clear that higher concentrations of FK506, including levels used in prior rodent work, can have a range of adverse effects in both rodents and humans, particularly nephrotoxicity [87,91]. It is intriguing to note that in spite of no quantifiable decrease in NFAT activity or brain cytokine levels, the concentrations of both FK506 and tat-VIVIT peptide still significantly decreased microgliosis and plaque load in the mice. We recognize that our method of indirectly assessing NFATc2 activity changes based upon changes in phosphorylation via Western blot is not the most sensitive or reliable means of assessing changes in binding ability. Future work employing electromobility shift assays would undoubtedly provide a clearer indication of changes in activity in both the periphery and the brain. We speculate that one possibility for the decrease in plaque load was simply due to improved clearance from the brain via increased microglial phagocytic ability. This suggests the provocative possibility that the modest changes in peripheral cytokine levels and presumably NFAT inhibition are enough to produce dramatic and significant effects on the brain. A further extension of this idea would be to determine whether modulation of peripheral NFAT activity and resultant cytokine or immune cell behavior is sufficient to limit brain inflammation. It is even feasible that peripheral NFAT inhibition is sufficient to limit infiltration of blood-derived immune cells into the brain, thus minimizing any exacerbation of an inflammatory response provided by the influxing peripheral leukocytes. In fact, NFATc2 has been shown in prior work to be particularly important in regulating glioblastoma invasion [85]. A strategy of peripheral NFAT targeting may eliminate or minimize the need for specific brain anti-inflammatory drugs to actually require brain penetration.

Regardless of the precise mechanism by which FK506 or tat-VIVIT treatment led to reduction in microgliosis and plaque load, it is still surprising that this did not result in improved performance via T-maze testing. It is possible that the testing paradigm we employed was not sufficiently sensitive enough to detect behavioral changes that result from the modest decrease in plaque load we observed. Additional memory testing protocols and more prolonged or robust drug regimens may provide changes that are quantifiable in the mouse model.

## Conclusions

These data support the idea that Aβ-mediated microgliosis and cytokine secretion is regulated, in part, by NFAT activity. Further studies to determine the appropriate dosage and treatment paradigms for FK506 and tat-VIVIT as well as to identify novel, brain penetrant NFAT inhibitors may provide a therapeutic approach for limiting the inflammatory component of AD.

## Additional file

Additional file 1: Figure S1. FK506 and tat-VIVIT did not alter brain protein levels or active NFATc2 levels in APP/PS1 mice. Twelve-month-old male APP/PS1 mice were treated via subcutaneous delivery for 28 days with no treatment (control), vehicle DMSO, 1 mg/kg/day FK506, 0.5 mg/kg/day VIVIT, or 0.5 mg/kg/day negative control scrambled peptide, VEET (*n* = 4/condition). Temporal cortices were collected, lysed, and separated by SDS-PAGE and Western blotted using anti-APP, BACE, PSD95, synaptophysin, pNFATc2, NFATc2 (loading control), anti-pNFκB (p65), anti-NFκB (p65) (loading control), c-Fos, and βIII tubulin (loading control) antibodies. Optical densities were normalized against their respective loading controls, averaged, and graphed (±SD).

## Abbreviations

AD: Alzheimer’s disease
Aβ: amyloid β
NFAT: nuclear factor of activated T cells
TNFα: tumor necrosis factor-α
APP: amyloid precursor protein
PSD95: post synaptic density protein 95
IL-1β: interleukin-1 beta
IL-6: interleukin-6
BACE: beta site APP cleaving enzyme
LPS: lipopolysaccharide

## References

[1] Dickson DW, Farlo J, Davies P, Crystal H, Fuld P, Yen SH (1988). Alzheimer’s disease. A double-labeling immunohistochemical study of senile plaques. Am J Pathol..

[2] Itagaki S, McGeer PL, Akiyama H, Zhu S, Selkoe D (1989). Relationship of microglia and astrocytes to amyloid deposits of Alzheimer disease. J Neuroimmunol.

[3] Miyazono M, Iwaki T, Kitamoto T, Kaneko Y, Doh-ura K, Tateishi J (1991). A comparative immunohistochemical study of Kuru and senile plaques with a special reference to glial reactions at various stages of amyloid plaque formation. Am J Pathol.

[4] Perlmutter LS, Barron E, Chui HC (1990). Morphologic association between microglia and senile plaque amyloid in Alzheimer’s disease. Neurosci Lett.

[5] Araujo DM, Cotman CW (1992). Beta-amyloid stimulates glial cells in vitro to produce growth factors that accumulate in senile plaques in Alzheimer’s disease. Brain Res.

[6] Bitting L, Naidu A, Cordell B, Murphy GM (1996). Beta-amyloid peptide secretion by a microglial cell line is induced by beta-amyloid-(25–35) and lipopolysaccharide. J Biol Chem.

[7] Davis JB, McMurray HF, Schubert D (1992). The amyloid beta-protein of Alzheimer’s disease is chemotactic for mononuclear phagocytes. Biochem Biophys Res Commun.

[8] Dhawan G, Floden AM, Combs CK (2012). Amyloid-beta oligomers stimulate microglia through a tyrosine kinase dependent mechanism. Neurobiol Aging.

[9] Haga S, Ikeda K, Sato M, Ishii T (1993). Synthetic Alzheimer amyloid beta/A4 peptides enhance production of complement C3 component by cultured microglial cells. Brain Res.

[10] Klegeris A, Walker DG, McGeer PL (1994). Activation of macrophages by Alzheimer beta amyloid peptide. Biochem Biophys Res Commun.

[11] Korotzer AR, Pike CJ, Cotman CW (1993). Beta-amyloid peptides induce degeneration of cultured rat microglia. Brain Res.

[12] Korotzer AR, Watt J, Cribbs D, Tenner AJ, Burdick D, Glabe C (1995). Cultured rat microglia express C1q and receptor for C1q: implications for amyloid effects on microglia. Exp Neurol.

[13] Korotzer AR, Whittemore ER, Cotman CW (1995). Differential regulation by beta-amyloid peptides of intracellular free Ca2+ concentration in cultured rat microglia. Eur J Pharmacol.

[14] Lorton D, Kocsis JM, King L, Madden K, Brunden KR (1996). Beta-amyloid induces increased release of interleukin-1 beta from lipopolysaccharide-activated human monocytes. J Neuroimmunol.

[15] McDonald DR, Brunden KR, Landreth GE (1997). Amyloid fibrils activate tyrosine kinase-dependent signaling and superoxide production in microglia. J Neurosci Off J Soc Neurosci.

[16] Meda L, Baron P, Prat E, Scarpini E, Scarlato G, Cassatella MA (1999). Proinflammatory profile of cytokine production by human monocytes and murine microglia stimulated with beta-amyloid[25–35]. J Neuroimmunol.

[17] Meda L, Bernasconi S, Bonaiuto C, Sozzani S, Zhou D, Otvos L (1996). Beta-amyloid (25–35) peptide and IFN-gamma synergistically induce the production of the chemotactic cytokine MCP-1/JE in monocytes and microglial cells. J Immunol.

[18] Meda L, Bonaiuto C, Baron P, Otvos L, Rossi F, Cassatella MA (1996). Priming of monocyte respiratory burst by beta-amyloid fragment (25–35). Neurosci Lett.

[19] Meda L, Cassatella MA, Szendrei GI, Otvos L, Baron P, Villalba M (1995). Activation of microglial cells by beta-amyloid protein and interferon-gamma. Nature.

[20] Sondag CM, Dhawan G, Combs CK (2009). Beta amyloid oligomers and fibrils stimulate differential activation of primary microglia. J Neuroinflammation..

[21] Tan J, Town T, Paris D, Mori T, Suo Z, Crawford F (1999). Microglial activation resulting from CD40-CD40L interaction after beta-amyloid stimulation. Science.

[22] Wu HY, Hudry E, Hashimoto T, Kuchibhotla K, Rozkalne A, Fan Z (2010). Amyloid beta induces the morphological neurodegenerative triad of spine loss, dendritic simplification, and neuritic dystrophies through calcineurin activation. J Neurosci Off J Soc Neurosci.

[23] Wu HY, Hudry E, Hashimoto T, Uemura K, Fan ZY, Berezovska O (2012). Distinct dendritic spine and nuclear phases of calcineurin activation after exposure to amyloid-beta revealed by a novel fluorescence resonance energy transfer assay. J Neurosci Off J Soc Neurosci.

[24] Brown DR, Herms JW, Schmidt B, Kretzschmar HA (1997). PrP and beta-amyloid fragments activate different neurotoxic mechanisms in cultured mouse cells. Eur J Neurosci.

[25] Combs CK, Johnson DE, Cannady SB, Lehman TM, Landreth GE (1999). Identification of microglial signal transduction pathways mediating a neurotoxic response to amyloidogenic fragments of beta-amyloid and prion proteins. J Neurosci Off J Soc Neurosci.

[26] Lorton D (1997). Beta-amyloid-induced IL-1 beta release from an activated human monocyte cell line is calcium- and G-protein-dependent. Mech Ageing Dev.

[27] Silei V, Fabrizi C, Venturini G, Salmona M, Bugiani O, Tagliavini F (1999). Activation of microglial cells by PrP and beta-amyloid fragments raises intracellular calcium through L-type voltage sensitive calcium channels. Brain Res.

[28] Goodman Y, Mattson MP (1994). Staurosporine and K-252 compounds protect hippocampal neurons against amyloid beta-peptide toxicity and oxidative injury. Brain Res.

[29] Copani A, Bruno V, Battaglia G, Leanza G, Pellitteri R, Russo A (1995). Activation of metabotropic glutamate receptors protects cultured neurons against apoptosis induced by beta-amyloid peptide. Mol Pharmacol.

[30] Le WD, Colom LV, Xie WJ, Smith RG, Alexianu M, Appel SH (1995). Cell death induced by beta-amyloid 1–40 in MES 23.5 hybrid clone: the role of nitric oxide and NMDA-gated channel activation leading to apoptosis. Brain Res.

[31] Smith-Swintosky VL, Zimmer S, Fenton JW, Mattson MP (1995). Opposing actions of thrombin and protease nexin-1 on amyloid beta-peptide toxicity and on accumulation of peroxides and calcium in hippocampal neurons. J Neurochem.

[32] Barger SW, Horster D, Furukawa K, Goodman Y, Krieglstein J, Mattson MP (1995). Tumor necrosis factors alpha and beta protect neurons against amyloid beta-peptide toxicity: evidence for involvement of a kappa B-binding factor and attenuation of peroxide and Ca2+ accumulation. Proc Natl Acad Sci U S A.

[33] Mattson MP, Tomaselli KJ, Rydel RE (1993). Calcium-destabilizing and neurodegenerative effects of aggregated beta-amyloid peptide are attenuated by basic FGF. Brain Res.

[34] Weiss JH, Pike CJ, Cotman CW (1994). Ca2+ channel blockers attenuate beta-amyloid peptide toxicity to cortical neurons in culture. J Neurochem.

[35] Kuchibhotla KV, Goldman ST, Lattarulo CR, Wu HY, Hyman BT, Bacskai BJ (2008). Abeta plaques lead to aberrant regulation of calcium homeostasis in vivo resulting in structural and functional disruption of neuronal networks. Neuron.

[36] Liu F, Grundke-Iqbal I, Iqbal K, Oda Y, Tomizawa K, Gong CX (2005). Truncation and activation of calcineurin A by calpain I in Alzheimer disease brain. J Biol Chem.

[37] Shankar GM, Bloodgood BL, Townsend M, Walsh DM, Selkoe DJ, Sabatini BL (2007). Natural oligomers of the Alzheimer amyloid-beta protein induce reversible synapse loss by modulating an NMDA-type glutamate receptor-dependent signaling pathway. J Neurosci Off J Soc Neurosci.

[38] Abdul HM, Sama MA, Furman JL, Mathis DM, Beckett TL, Weidner AM (2009). Cognitive decline in Alzheimer’s disease is associated with selective changes in calcineurin/NFAT signaling. J Neurosci Off J Soc Neurosci.

[39] Celsi F, Svedberg M, Unger C, Cotman CW, Carri MT, Ottersen OP (2007). Beta-amyloid causes downregulation of calcineurin in neurons through induction of oxidative stress. Neurobiol Dis.

[40] Dineley KT, Hogan D, Zhang WR, Taglialatela G (2007). Acute inhibition of calcineurin restores associative learning and memory in Tg2576 APP transgenic mice. Neurobiol Learn Mem.

[41] Hudry E, Wu HY, Arbel-Ornath M, Hashimoto T, Matsouaka R, Fan Z (2012). Inhibition of the NFAT pathway alleviates amyloid beta neurotoxicity in a mouse model of Alzheimer’s disease. J Neurosci Off J Soc Neurosci.

[42] Norris CM, Kadish I, Blalock EM, Chen KC, Thibault V, Porter NM (2005). Calcineurin triggers reactive/inflammatory processes in astrocytes and is upregulated in aging and Alzheimer’s models. J Neurosci Off J Soc Neurosci.

[43] Oh-hora M, Rao A (2009). The calcium/NFAT pathway: role in development and function of regulatory T cells. Microbes Infect.

[44] Benedito AB, Lehtinen M, Massol R, Lopes UG, Kirchhausen T, Rao A (2005). The transcription factor NFAT3 mediates neuronal survival. J Biol Chem.

[45] Graef IA, Wang F, Charron F, Chen L, Neilson J, Tessier-Lavigne M (2003). Neurotrophins and netrins require calcineurin/NFAT signaling to stimulate outgrowth of embryonic axons. Cell.

[46] Luoma JI, Zirpel L (2008). Deafferentation-induced activation of NFAT (nuclear factor of activated T-cells) in cochlear nucleus neurons during a developmental critical period: a role for NFATc4-dependent apoptosis in the CNS. J Neurosci Off J Soc Neurosci.

[47] Pérez-Ortiz JM, Serrano-Pérez MC, Pastor MD, Martín ED, Calvo S, Rincón M (2008). Mechanical lesion activates newly identified NFATc1 in primary astrocytes: implication of ATP and purinergic receptors. Eur J Neurosci.

[48] Sama MA, Mathis DM, Furman JL, Abdul HM, Artiushin IA, Kraner SD (2008). Interleukin-1beta-dependent signaling between astrocytes and neurons depends critically on astrocytic calcineurin/NFAT activity. J Biol Chem.

[49] Shaw KT, Ho AM, Raghavan A, Kim J, Jain J, Park J (1995). Immunosuppressive drugs prevent a rapid dephosphorylation of transcription factor NFAT1 in stimulated immune cells. Proc Natl Acad Sci U S A..

[50] Macian F, Garcia-Rodriguez C, Rao A (2000). Gene expression elicited by NFAT in the presence or absence of cooperative recruitment of Fos and Jun. EMBO J.

[51] Masuda ES, Imamura R, Amasaki Y, Arai K, Arai N (1998). Signalling into the T-cell nucleus: NFAT regulation. Cell Signal.

[52] Rao A, Luo C, Hogan PG (1997). Transcription factors of the nfat family: regulation and function. Immunol.15.1.707. Annu Rev Immunol.

[53] Boise LH, Petryniak B, Mao X, June CH, Wang CY, Lindsten T (1993). The NFAT-1 DNA binding complex in activated T cells contains Fra-1 and JunB. Mol Cell Biol.

[54] Jain J, McCafffrey PG, Miner Z, Kerppola TK, Lambert JN, Verdine GL (1993). The T-cell transcription factor NFATp is a substrate for calcineurin and interacts with Fos and Jun. Nature..

[55] Yang XY, Wang LH, Chen T, Hodge DR, Resau JH, DaSilva L (2000). Activation of human T lymphocytes is inhibited by peroxisome proliferator-activated receptor gamma (PPARgamma) agonists. PPARgamma co-association with transcription factor NFAT. J Biol Chem..

[56] Fisher WG, Yang PC, Medikonduri RK, Jafri MS (2006). NFAT and NFkappaB activation in T lymphocytes: a model of differential activation of gene expression. Ann Biomed Eng.

[57] Bao Y, Li R, Jiang J, Cai B, Gao J, Le K (2008). Activation of peroxisome proliferator-activated receptor gamma inhibits endothelin-1-induced cardiac hypertrophy via the calcineurin/NFAT signaling pathway. Mol Cell Biochem.

[58] Putt ME, Hannenhalli S, Lu Y, Haines P, Chandrupatla HR, Morrisey EE (2009). Evidence for coregulation of myocardial gene expression by MEF2 and NFAT in human heart failure. Circ Cardiovasc Genet.

[59] Shaw JP, Utz P, Durand DB, Toole JJ, Emmel EA, Crabtree GR (1988). Identification of a putative regulator of early T cell activation genes. Science.

[60] Shioda N, Han F, Moriguchi S, Fukunaga K (2007). Constitutively active calcineurin mediates delayed neuronal death through Fas-ligand expression via activation of NFAT and FKHR transcriptional activities in mouse brain ischemia. J Neurochem.

[61] Canellada A, Ramirez BG, Minami T, Redondo JM, Cano E (2008). Calcium/calcineurin signaling in primary cortical astrocyte cultures: Rcan1-4 and cyclooxygenase-2 as NFAT target genes. Glia.

[62] Dever SM, Xu R, Fitting S, Knapp PE, Hauser KF (2012). Differential expression and HIV-1 regulation of mu-opioid receptor splice variants across human central nervous system cell types. J Neurovirol.

[63] Furman JL, Sama DM, Gant JC, Beckett TL, Murphy MP, Bachstetter AD (2012). Targeting astrocytes ameliorates neurologic changes in a mouse model of Alzheimer’s disease. J Neurosci Off J Soc Neurosci.

[64] Jones EA, Sun D, Kobierski L, Symes AJ (2003). NFAT4 is expressed in primary astrocytes and activated by glutamate. J Neurosci Res.

[65] Kim B, Jeong HK, Kim JH, Lee SY, Jou I, Joe EH (2011). Uridine 5′-diphosphate induces chemokine expression in microglia and astrocytes through activation of the P2Y6 receptor. J Immunol.

[66] Serrano-Perez MC, Martin ED, Vaquero CF, Azcoitia I, Calvo S, Cano E (2011). Response of transcription factor NFATc3 to excitotoxic and traumatic brain insults: identification of a subpopulation of reactive astrocytes. Glia.

[67] Luoma JI, Zirpel L (2008). Deafferentation-induced activation of NFAT (nuclear factor of activated T-cells) in cochlear nucleus neurons during a developmental critical period: a role for NFATc4-dependent apoptosis in the CNS. J Neurosci.

[68] Ferrari D, Stroh C, Schulze-Osthoff K, Ferrari D, Stroh C, Schulze-Osthoff K (1999). P2X7/P2Z purinoreceptor-mediated activation of transcription factor NFAT in microglial cells. J Biol Chem.

[69] Kataoka A, Tozaki-Saitoh H, Koga Y, Tsuda M, Inoue K (2009). Activation of P2X7 receptors induces CCL3 production in microglial cells through transcription factor NFAT. J Neurochem.

[70] Nagamoto-Combs K, Combs CK (2010). Microglial phenotype is regulated by activity of the transcription factor, NFAT (nuclear factor of activated T cells). J Neurosci Off J Soc Neurosci.

[71] Rojanathammanee L, Puig KL, Combs CK (2013). Pomegranate polyphenols and extract inhibit nuclear factor of activated T-cell activity and microglial activation in vitro and in a transgenic mouse model of Alzheimer disease. J Nutr.

[72] Shiratori M, Tozaki-Saitoh H, Yoshitake M, Tsuda M, Inoue K (2010). P2X7 receptor activation induces CXCL2 production in microglia through NFAT and PKC/MAPK pathways. J Neurochem.

[73] Aramburu J, Yaffe MB, Lopez-Rodriguez C, Cantley LC, Hogan PG, Rao A (1999). Affinity-driven peptide selection of an NFAT inhibitor more selective than cyclosporin A. Science.

[74] Noguchi H, Matsushita M, Okitsu T, Moriwaki A, Tomizawa K, Kang S (2004). A new cell-permeable peptide allows successful allogeneic islet transplantation in mice. Nat Med.

[75] Kuriyama M, Matsushita M, Tateishi A, Moriwaki A, Tomizawa K, Ishino K (2006). A cell-permeable NFAT inhibitor peptide prevents pressure-overload cardiac hypertrophy. Chem Biol Drug Des.

[76] Brooks H, Lebleu B, Vivès E (2005). Tat peptide-mediated cellular delivery: back to basics. Adv Drug Deliv Rev Protein Pept-Med Transduction: Mech Implications Drug Deliv.

[77] Vives E (2005). Present and future of cell-penetrating peptide mediated delivery systems: “Is the Trojan horse too wild to go only to Troy?”. J Controlled Release..

[78] Henderson DJ, Naya I, Bundick RV, Smith GM, Schmidt JA (1991). Comparison of the effects of FK-506, cyclosporin A and rapamycin on IL-2 production. Immunology.

[79] Gonzalez-Pinto IM, Rimola A, Margarit C, Cuervas-Mons V, Abradelo M, Alvarez-Laso C (2005). Five-year follow-up of a trial comparing tacrolimus and cyclosporine microemulsion in liver transplantation. Transplant Proc.

[80] Dhawan G, Combs CK (2012). Inhibition of Src kinase activity attenuates amyloid associated microgliosis in a murine model of Alzheimer’s disease. J Neuroinflammation..

[81] Bradford MM (1976). A rapid and sensitive method for the quantitation of microgram quantities of protein utilizing the principle of protein-dye binding. Anal Biochem..

[82] Wenk GL. Assessment of spatial memory using the T maze. Curr Protoc Neurosci. 1998;Chapter 8:Unit 8 5B. 10.1002/0471142301.ns0805bs04 18428543

[83] Vihma H, Pruunsild P, Timmusk T (2008). Alternative splicing and expression of human and mouse NFAT genes. Genomics.

[84] Floden AM, Li S, Combs CK (2005). {Beta}-amyloid-stimulated microglia induce neuron death via synergistic stimulation of tumor necrosis factor {alpha} and NMDA receptors. J Neurosci.

[85] Tie X, Han S, Meng L, Wang Y, Wu A (2013). NFAT1 is highly expressed in, and regulates the invasion of, glioblastoma multiforme cells. PLoS One.

[86] Liu J, Farmer JD, Lane WS, Friedman J, Weissman I, Schreiber SL (1991). Calcineurin is a common target of cyclophilin-cyclosporin A and FKBP-FK506 complexes. Cell.

[87] Su Q, Weber L, Le Hir M, Zenke G, Ryffel B (1995). Nephrotoxicity of cyclosporin A and FK506: inhibition of calcineurin phosphatase. Ren Physiol Biochem.

[88] Rozkalne A, Hyman BT, Spires-Jones TL (2011). Calcineurin inhibition with FK506 ameliorates dendritic spine density deficits in plaque-bearing Alzheimer model mice. Neurobiol Dis.

[89] Spires-Jones TL, Kay K, Matsouka R, Rozkalne A, Betensky RA, Hyman BT (2011). Calcineurin inhibition with systemic FK506 treatment increases dendritic branching and dendritic spine density in healthy adult mouse brain. Neurosci Lett.

[90] Taglialatela G, Hogan D, Zhang WR, Dineley KT (2009). Intermediate- and long-term recognition memory deficits in Tg2576 mice are reversed with acute calcineurin inhibition. Behav Brain Res.

[91] Reding R, Wallemacq PE, Lamy ME, Rahier J, Sempoux C, Debande B (1994). Conversion from cyclosporine to FK506 for salvage of immunocompromised pediatric liver allografts. Efficacy, toxicity, and dose regimen in 23 children. Transplantation.

[92] Aramburu J, Garcia-Cozar F, Raghavan A, Okamura H, Rao A, Hogan PG (1998). Selective inhibition of NFAT activation by a peptide spanning the calcineurin targeting site of NFAT. Mol Cell.

[93] Yu H, van Berkel TJ, Biessen EA (2007). Therapeutic potential of VIVIT, a selective peptide inhibitor of nuclear factor of activated T cells, in cardiovascular disorders. Cardiovasc Drug Rev.

[94] Yoshiyama Y, Higuchi M, Zhang B, Huang SM, Iwata N, Saido TC (2007). Synapse loss and microglial activation precede tangles in a P301S tauopathy mouse model. Neuron.

