# Supplementary figures and images for: Attenuation of microglial activation in a mouse model of Alzheimer’s disease via NFAT inhibition
Source: J Neuroinflammation. 2015 Mar 4;12:42. doi: 10.1186/s12974-015-0255-2 (PMC4355356; doi:10.1186/s12974-015-0255-2)

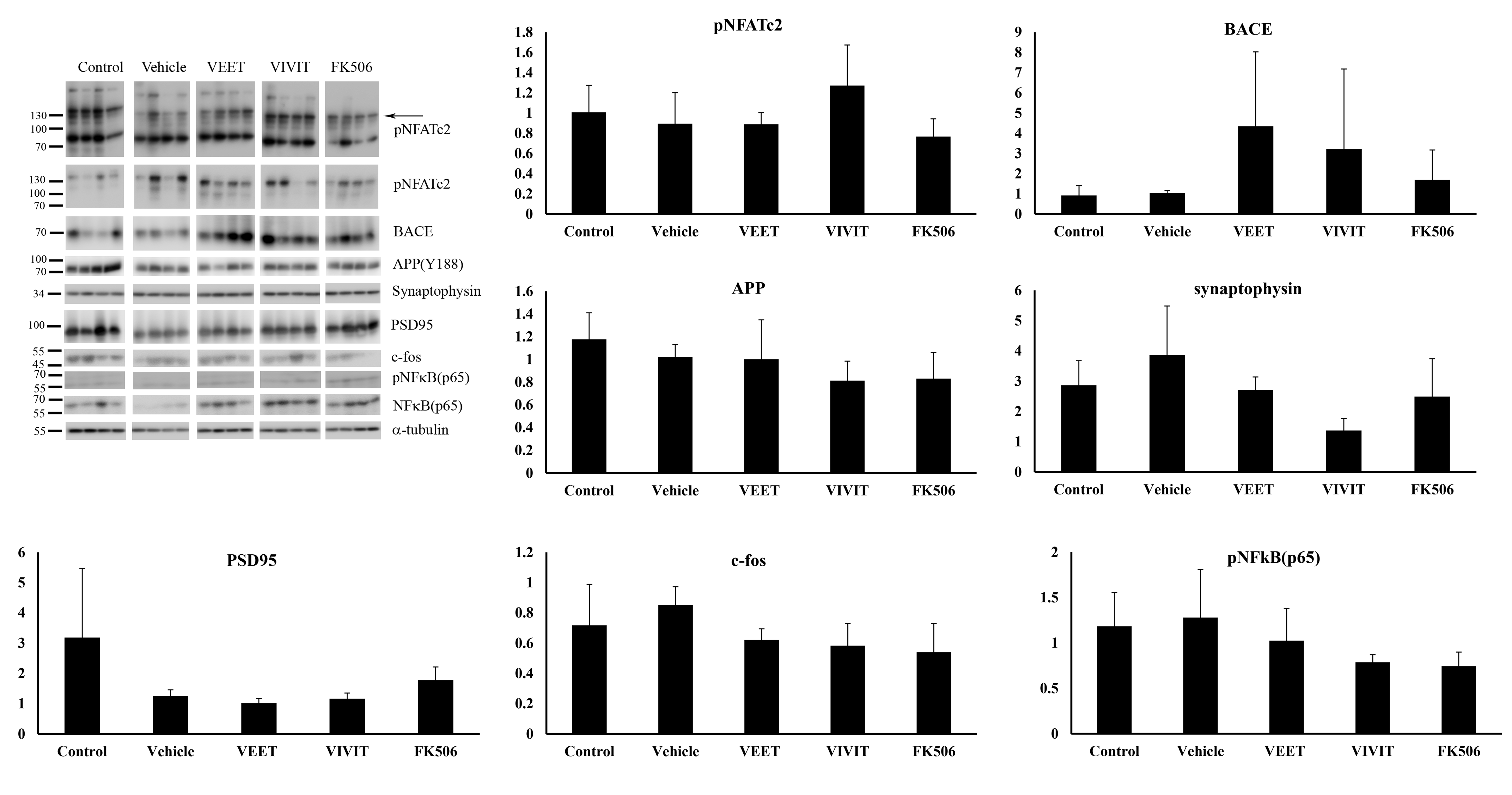

Supplement: Additional file 1: Figure S1. — FK506 and tat-VIVIT did not alter brain protein levels or active NFATc2 levels in APP/PS1 mice. Twelve-month-old male APP/PS1 mice were treated via subcutaneous delivery for 28 days with no treatment (control), vehicle DMSO, 1 mg/kg/day FK506, 0.5 mg/kg/day VIVIT, or 0.5 mg/kg/day negative control scrambled peptide, VEET (n = 4/condition). Temporal cortices were collected, lysed, and separated by SDS-PAGE and Western blotted using anti-APP, BACE, PSD95, synaptophysin, pNFATc2, NFATc2 (loading control), anti-pNFκB (p65), anti-NFκB (p65) (loading control), c-Fos, and βIII tubulin (loading control) antibodies. Optical densities were normalized against their respective loading controls, averaged, and graphed (±SD). [file 12974_2015_255_MOESM1_ESM.tiff]
